# Assessing the Effectiveness of Selective RET Inhibitors in RET-Positive Cancers through Fluorodeoxyglucose Uptake Analysis

**DOI:** 10.3390/diagnostics14171886

**Published:** 2024-08-28

**Authors:** Kalevi Kairemo, Homer A. Macapinlac, Mohammed Gouda, Vivek Subbiah

**Affiliations:** 1 Department of Nuclear Medicine, The University of Texas MD Anderson Cancer Center, Houston, TX 77030, USA; 2 Department of Investigational Cancer Therapeutics, Division of Cancer Medicine, The University of Texas MD Anderson Cancer Center, Houston, TX 77030, USA; 3 Sarah Cannon Research Institute, Nashville, TN 37203, USA; vivek.subbiah@scri.com

**Keywords:** fluoro-18 deoxyglucose (^18^F-FDG), positron emission tomography FDG, computerized tomography (PET/CT), molecular imaging, tyrosine kinase receptors, *ret* mutation, response evaluation, targeted therapy

## Abstract

Selective RET inhibitors, such as selpercatinib and pralsetinib, have revolutionized the treatment of cancers with RET gene alterations. These inhibitors have shown remarkable clinical efficacy, particularly in RET-driven lung cancer, medullary thyroid cancer, and other solid tumors driven by RET gene fusions. The assessment of treatment response in oncology has been greatly enhanced by Fluorodeoxyglucose Positron Emission Tomography (FDG-PET), a valuable tool that measures tumor metabolism and provides early indicators of treatment effectiveness. This work explores the effectiveness of selective RET inhibitors in targeting RET-positive cancers and investigates the utility of FDG-PET in assessing treatment response. The paper includes insightful case studies that highlight the successful application of RET inhibitors in the treatment of RET-positive cancers. The findings suggest that FDG-PET has the potential to serve as a non-invasive biomarker for monitoring treatment response in patients with RET-positive cancers. However, further research is required to establish standardized criteria for interpreting FDG-PET scans in the context of selective RET inhibitors and to uncover the broader applications of FDG-PET in precision oncology.

## 1. Introduction

In recent years, targeted kinase inhibitors have gained significant prominence in the management of advanced medullary thyroid cancer (MTC), lung cancer, and various other malignancies [1]. Among these breakthrough treatments, the emergence of selective RET inhibitors, such as selpercatinib and pralsetinib, has revolutionized the therapeutic landscape for cancers characterized by RET gene alterations [1,2,3,4]. These inhibitors have been specifically designed to target and inhibit the abnormal activity of RET proteins in cancer cells, offering a promising avenue for treatment. RET, a proto-oncogene, plays a crucial role in the growth, differentiation, and survival of cells [2]. When RET becomes abnormally activated or fused with other genes, it can drive the development and progression of several types of cancers. This is where selective RET inhibitors come into play, as they effectively block RET signaling, thereby halting tumor growth and potentially inducing tumor regression [2].

The clinical efficacy of selective RET inhibitors in RET-positive cancers has been remarkable. Selpercatinib, in particular, has shown impressive results in clinical trials, leading to its approval for multiple indications by the FDA [5,6]. It has demonstrated significant effectiveness in RET fusion-positive lung cancer and RET-mutated MTC, resulting in substantial tumor shrinkage and extended periods of progression-free survival for patients. Selpercatinib has also exhibited promising activity in other RET-driven malignancies, including RET fusion-positive thyroid cancer and other solid tumors with RET gene fusions [7].

Similarly, pralsetinib has shown notable activity in RET fusion-positive lung cancer and RET-mutant MTC [3]. Clinical studies have revealed substantial tumor responses and durable benefits in patients treated with pralsetinib, leading to its approval for these specific indications.

### The Role of FDG-PET in Assessing Response to Tyrosine Kinase Targeted Therapy and Its Potential Application in Selective RET Inhibitor Response Assessment

FDG-PET (Fluorodeoxyglucose Positron Emission Tomography) is a widely utilized imaging modality in oncology for assessing tumor metabolism and response to therapy [8]. This imaging technique relies on the principle that cancer cells, due to their high metabolic activity, have an increased uptake of glucose, which is visualized as increased FDG uptake on PET scans. In the context of tyrosine kinase targeted therapy, FDG-PET has proven to be a valuable tool for assessing treatment response [9,10]. Tyrosine kinase inhibitors (TKIs) selectively target specific signaling pathways involved in tumor growth and survival [11]. However, the response to TKIs can vary among patients, and early identification of treatment response is crucial for optimizing patient care. FDG-PET can provide valuable information on the metabolic changes within the tumor following TKI treatment. Changes in FDG uptake on PET scans can indicate alterations in tumor metabolism and can potentially serve as an early marker of treatment response. A reduction in FDG uptake, known as metabolic response, has been associated with favorable treatment outcomes, including improved progression-free survival and overall survival in various malignancies. In the specific context of selective RET inhibitors, the role of FDG-PET in response assessment is an area of ongoing research. RET gene alterations, such as fusions or mutations, drive the oncogenic signaling in RET-positive cancers. Selective RET inhibitors, such as selpercatinib and pralsetinib, specifically target these RET alterations and have shown promising clinical efficacy [4]. FDG-PET may have a potential role in assessing responses to selective RET inhibitors. Early studies have shown that changes in FDG uptake on PET scans correlate with treatment response in RET-positive cancers treated with multi-kinase inhibitors that have RET activity like lenvatinib or cabozantinib or vandetanib [12,13,14]. In this work, we explore the efficacy of selective RET inhibitors targeting RET-positive cancers, employing FDG uptake analysis as our primary investigative tool. Additionally, we present compelling case studies showcasing the application of RET inhibitors in the treatment of patients afflicted with RET-positive cancers.

## 2. Detailed Case Descriptions

Case 1: A male in his 50s with medullary thyroid carcinoma was diagnosed twelve years earlier and treated initially with a total thyroidectomy and surgical removal of 34 lymph nodes and a 1 cm medullary thyroid cancer with extrathyroidal extension and lymphovascular invasion. There was C-cell hyperplasia. There were lymph node metastases in 8 of 34 lymph nodes and the largest focus was 3 cm. There was extranodal extension present. He subsequently underwent a second procedure for left parapharyngeal space-occupying lesions that had the potential for metastatic disease five months later. The mass was resected and documented as a metastatic medullary thyroid carcinoma. Postoperatively, the patient did well, and 2 days after surgery, he had a serum calcitonin of 88.5 pg/mL and a serum CEA of 5.3 ng/mL.

**Figure 1:**
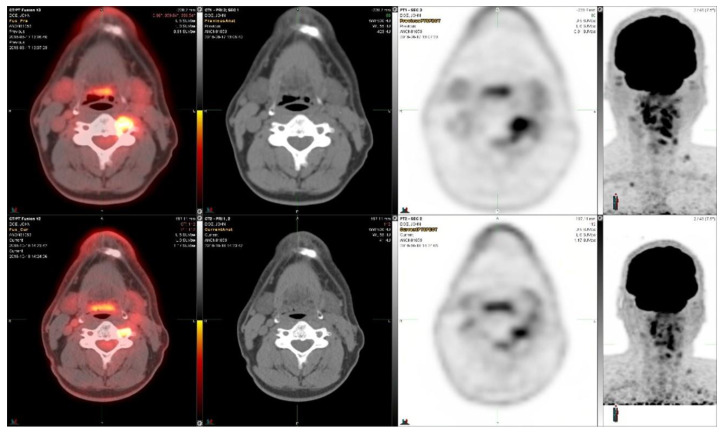
^18^F-FDG-PET/CT images from a medullary thyroid cancer patient who had an RET mutation and was treated with selpercanitib for almost five years. The upper panel demonstrates the situation before selpercanitib treatment; from the left, transaxial PET/CT fusion images, CT images, and PET images at the level of bone metastases (C2–C3), and the furthest right image shows the MIP image from the PET study on the head and neck region. The lower panel shows the situation after two cycles of selpercanitib two months later through similar images at the same level in the transaxial images and in the MIP image shown furthest to the right. In the bone metastasis, the SUVmax decreased from 7.9 to 6.2, corresponding to a 22% decrease.

Two years later he was treated with a total dose of 45 Gy in 15 fractions using an IMRT radiotherapy technique for C2-C3 lesions in the cervical spine. He was followed for 3 years when his tumor markers started to rise. Based on his RET alteration, he was enrolled on the selpercanitib (RET inhibitor) study [3]. He received a total of 37 cycles (160 mg orally BID). Clinically, he appears to have tolerated therapy well with no significant adverse events. The response to the *RET*-inhibitor treatment is shown in Figure 1.

The early response this RET-inhibitor treatment in the first two months is shown in Figure 1. The SUVmax decreased from 7.9 to 6.2, i.e., −22%, in the bone metastasis shown in Figure 1. The tumor size decreased in this metastasis from 1.2 cm × 1.1 cm to 1.1 cm × 1.0 cm. The SUVmax in the largest lymph node decreased from 8.1 to 6.4, i.e., −21%, whereas the tumor size decreased in this metastasis from 1.8 cm × 1.5 cm to 1.4 cm × 1.0 cm.

**Figure 2:**
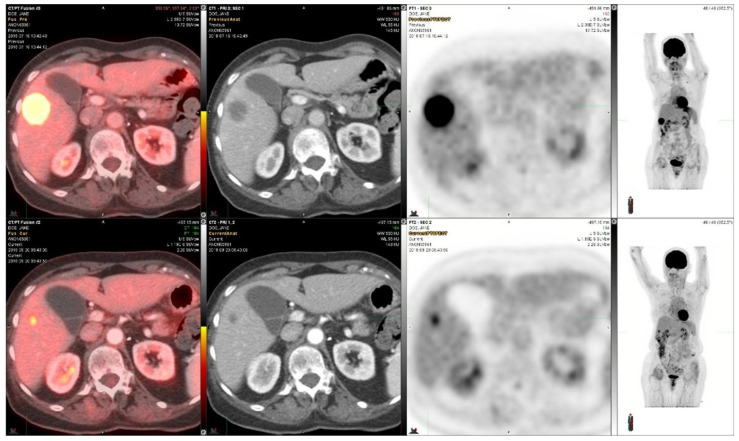
^18^F-FDG-PET/CT images in a patient with metastatic adenocarcinoma of the lung which had an RET-mutation and was treated with selpercanitib for 12 cycles. The upper panel demonstrates the situation before selpercanitib treatment; from the left, transaxial PET/CT fusion images, CT images, and PET images at the level of the largest liver metastasis and the furthest right image shows n MIP image from the whole-body PET study. The lower panel shows the situation after two cycles of selpercanitib two months later using images at the same level in the transaxial images and in the MIP image shown furthest to the right. In this liver metastasis, the SUVmax decreased from 21.0 to 5.2, corresponding to a 75% decrease.

Case 2: A female in her 70s was diagnosed with stage IV metastatic adenocarcinoma of the lung five years earlier. The initial treatment consisted of weekly paclitaxel in combination with carboplatin for two months and mediastinal external beam radiation therapy. Genomic testing revealed an RET fusion-positive lung cancer. Based on this, the patient was started oral vandetinib, a multi-kinase VEGF inhibitor with RET activity, and everolimus, an mTOR inhibitor, which were to be taken daily for twelve cycles and thereafter every other day based on a clinical trial [15]. Following progression of the disease, the patient was started on selpercanitib (an RET inhibitor). The initial PET/CT scan showed activity in the liver. A subsequent scan at re-staging after two cycles showed a response in the liver metastasis (Figure 2). The SUVmax decreased from 21.0 to 5.2, i.e., −75%, in the liver metastasis shown in Figure 2. The liver tumor size decreased from 3.1 cm × 2.8 cm to 0.9 cm × 0.8 cm. She received a total of 12 cycles and progressed on therapy. The overall survival of stage IV metastatic lung adenocarcinoma is more than five years.

Case 3: A 28-year-old female presented with a right pleural mass on biopsy—identified as adenocarcinoma—eleven years ago and she was originally treated with palliative chemotherapy consisting of pemetrexed and carboplatin. Avastin was also added in the second cycle. The patient received a total of six cycles of treatment.

Next, she underwent a right extra-pleural pneumonectomy with resection and reconstruction of the diaphragm and pericardium on the right and mediastinal lymph node dissection. A histological review of the surgical specimen revealed a poorly differentiated adenocarcinoma involving the lung parenchyma, diaphragm, pericardium, and chest wall. There was evidence of extensive lymphovascular invasion. Two hilar lymph nodes as well as several mediastinal lymph nodes were positive for metastatic adenocarcinoma including one paraesophageal lymph node, seven lymph nodes in four out of thirteen possible locations, one of two 4R lymph nodes, the right paratracheal nodes, and two of three lymph nodes in the apical tissue, as well as the second and sixth intercostal space lymph nodes.

The patient made a good recovery from surgery and then proceeded to receive adjuvant intensity modulated proton beam radiotherapy of 50 Gy in 25 fractions. Three years later the patient consented to participating in a clinical trial consisting of vandetinib and everolimus [15]. After two and a half years she was taken off protocol due to progression. A baseline PET CT showed cancer in multiple areas in the upper abdominal region. The patient was initiated on an *RET*-targeted therapy with selpercatinib. The patient was on selpercatinib therapy for almost five years, deriving clinical benefit and a complete metabolic response. However, her tumors progressed and she was taken off the RET inhibitor.

**Figure 3:**
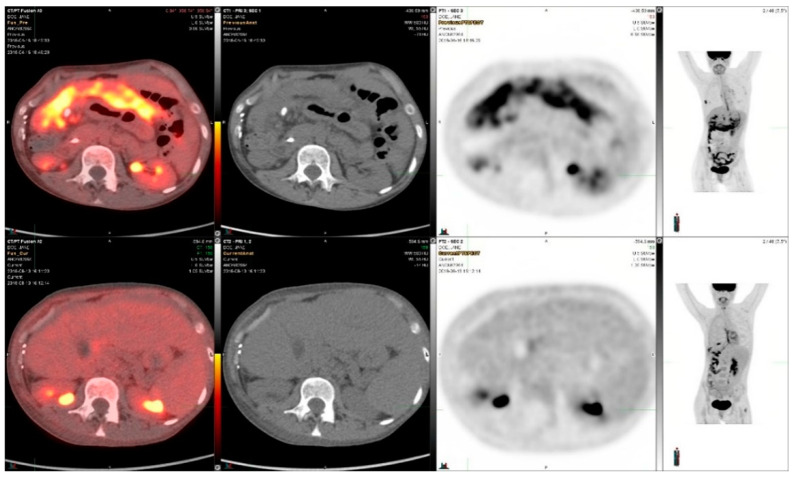
^18^F-FDG-PET/CT images in a patient with metastatic adenocarcinoma of the pleura which had an RET-mutation and was treated with selpercanitib for 6 cycles. The upper panel demonstrates the situation before selpercanitib treatment; from the left, transaxial PET/CT fusion images, CT images, and PET images in the upper abdominal region and the furthest right image shows an MIP image from the whole-body PET study. The lower panel shows the situation after two cycles of selpercanitib two months later in images at the same level in the transaxial images and in the MIP image furthest to the right. In this metastatic area the SUVmax decreased from 8.2 to 2.1, corresponding to a 74% decrease.

Figure 3 demonstrates a response in the upper abdominal region after two cycles in the beginning of ret targeted therapy. The SUVmax decreased from 8.2 to 2.1, i.e., −74%, in the metastatic area shown in Figure 3.

## 3. Discussion

A reduction in FDG uptake after treatment initiation has been associated with improved clinical outcomes, suggesting that FDG-PET can serve as a non-invasive biomarker for monitoring treatment responses in this patient population. Furthermore, FDG-PET may have a complementary role alongside other conventional imaging modalities, such as computed tomography (CT) scans, in the assessment of treatment response. While CT scans primarily evaluate anatomical changes, FDG-PET provides functional information about tumor metabolism. Combining these imaging modalities can potentially provide a more comprehensive assessment of treatment response and aid in treatment decision making. However, further research is needed to establish standardized criteria for interpreting FDG-PET scans in the context of selective RET inhibitors.

The use of selective RET inhibitors offers several advantages for the treatment of RET-positive cancers. Firstly, these inhibitors provide a targeted approach tailored to the specific molecular abnormalities driving the cancer, offering improved outcomes and reduced toxicity compared to traditional chemotherapy. Additionally, selective RET inhibitors have shown efficacy even in patients who have previously progressed on systemic therapies, providing a new treatment option for those with limited alternatives. The introduction of selective RET inhibitors has truly transformed the management of RET-positive cancers, offering a targeted and effective treatment option for patients with these specific molecular alterations. The remarkable clinical responses observed in RET fusion-positive lung cancer, RET-mutant MTC, and other RET-driven malignancies highlight the potential of these inhibitors to significantly improve outcomes for patients facing these challenging cancers.

*All three of our patients demonstrated a response to selpercatinib* {6-(2-hydroxy-2-methylpropoxy)-4-[6-[6-[(6-methoxypyridin-3-yl)methyl]-3,6-diazabicyclo[3.1.1]heptan-3-yl]pyridin-3-yl]pyrazolo[1,5-a]pyridine-3-carbonitrile}, which is a new highly sensitive RET kinase inhibitor that was evaluated by PET CT scans.

Vandetanib, a multi-kinase inhibitor with RET kinase inhibitory activity, has been enlisted against medullary thyroid cancer. Within a PET study encompassing 18 MTC cases, a somatic RET mutation emerged in three out of eight potential patients, yet the separate FDG data for the ret-fusion positive patients remained undisclosed [16]. Werner et al. reported that, within ^18^F-FDG-PET/CT scans, the most metabolically active lesion could prognosticate progression-free survival in MTC treated with vandetanib, although not overall survival [17]. Their investigation extended to intratumoral radiomic features and PET parameters, revealing that only total lesion glycolysis (TLG) could discern low- and high-risk patients. In a recent study by Bardet et al. [18], two patients diagnosed with medullary thyroid cancer were treated with selpercatinib, a highly selective RET kinase inhibitor. These patients exhibited a remarkable decrease in calcitonin levels, with both experiencing a 99% reduction. One patient had extensive lymph node and lung metastases, while the other had diffuse bone and liver disease. Objective tumor responses were observed in both cases, with significant clinical improvement and an RECIST 1.1 response based on tumor size measurements, reaching up to a 90% reduction in the younger patient. However, an intriguing finding emerged during treatment with selpercatinib—both patients experienced a gradual and sustained increase in CEA levels [17]. In fact, the levels rose by 207% and 835%, respectively. Surprisingly, the ^18^FDG PET/CT scans did not reveal any abnormal uptake that could explain this elevation in CEA. This discrepancy prompted further investigation into the use of FDG-PET as a potential surrogate marker for response assessment in RET-positive cancers. RET fusions are known to be oncogenic in 1–2% of non-small cell lung cancers, and the clinical trials leading to FDA approval of selpercatinib primarily relied on RECIST criteria to evaluate tumor shrinkage. However, for patients with non-measurable disease, alternative assessment modalities are required. FDG-PET has been established as a reliable surrogate marker for response in various oncogene-driven cancers, although limited data exists for its use in RET-positive cancers. In light of this, the authors hypothesized that some RET-positive cancers could be evaluated using FDG-PET, allowing for a qualitative assessment of treatment response. In this report, the authors present three cases of advanced mutant RET medullary thyroid cancer or lung adenocarcinoma, where the tumor responses to selpercatinib were measured using 2-deoxy-2-(^18^F)fluoro-D-glucose (^18^FDG)-PET/CT. This novel approach revealed valuable insights into the treatment response of RET positive cancers. By utilizing FDG-PET/CT scans, the authors were able to assess the metabolic changes within the tumors and evaluate the effectiveness of selpercatinib. This methodology provides an alternative means of evaluating treatment response in patients with non-measurable disease, offering a more comprehensive understanding of the therapeutic efficacy of selective RET kinase inhibitors. Overall, this case report highlights the potential of FDG-PET/CT as a valuable tool for response assessment in RET positive cancers treated with selpercatinib. Further research and larger studies are warranted to establish the utility of FDG-PET in this context and to explore its broader applications in precision oncology. Similarly sparse are reports concerning lung cancer and RET fusions and FDG PET.

Beyond lung and thyroid cancer, a recent case reported a patient with RET fusion-positive pleiomorphic sarcoma that responded to a neoadjuvant RET inhibitor and that the neoadjuvant reduction in tumor glucose activity and size was assessed by FDG PET [18]. In this patient with a locally advanced T2 tumor posing a high risk of metastasis, they opted for a dual approach utilizing selpercatinib as both a neoadjuvant and adjuvant treatment. The efficacy of the short-term neoadjuvant therapy became apparent remarkably quickly, with visible clinical improvement within just 3 days of treatment. By the 12th day, a notable reduction in both tumor volume and metabolic activity was evident according to FDG PET. Subsequent assessments at the 4-week mark revealed an even more impressive tumor shrinkage, amounting to a remarkable 90% reduction compared to the initial tumor volume. Ultimately, the sarcoma was successfully excised. The utilization of PET/CT guidance proved pivotal in this pioneering instance of employing neoadjuvant therapy for sarcoma.

Recent advancements spotlight the efficacy of selpercatinib, a selective RET inhibitor, particularly in non-small cell lung cancer and RET-positive thyroid cancer, even extending to a tissue agnostic approval [5,6]. Evidence from the LIBRETTO-001 trial underscores selpercatinib’s prowess, demonstrating promising outcomes across various tumor types with RET fusion-positive profiles. Given the burgeoning approvals and implications in the tissue agnostic arena, many patients with RET fusion positive cancers may present with non-measurable disease on plain CT scans. In such scenarios, FDG PET emerges as a viable avenue for assessing and monitoring patients undergoing treatment with selective RET inhibitors.

Preliminary insights gleaned from three cases hint at the potential of ^18^FDG PET/CT as a tool for evaluating disease control and metabolic response in RET-positive cancers. However, further rigorous investigations are imperative to unravel the full potential of FDG PET as both a qualitative and quantitative imaging modality in this domain.

## 4. Conclusions

In summary, FDG-PET plays a crucial role in assessing responses to tyrosine kinase targeted therapy. In the case of selective RET inhibitors, FDG-PET holds promise as a non-invasive imaging modality for monitoring treatment response. Incorporating FDG-PET into the assessment of selective RET inhibitor responses may provide valuable insights into the metabolic changes within tumors and aid in treatment decision making for patients with RET-positive cancers.

## Data Availability

The original contributions presented in the study are included in the article, further inquiries can be directed to the corresponding author.
